# Machine learning prediction of hypertension integrating polygenic risk scores in inner Eurasian populations

**DOI:** 10.3389/fcvm.2026.1843103

**Published:** 2026-07-08

**Authors:** Vera Tsvetkova, Aleksandra Denisova, Saleem Mansour, Layal Shaheen, Iskandar Hweijeh, Leushin Artem, Travin Grigorii, Dilya Turkmenova, Liya Valieva, Anna Kim, Dmitrii Kharitonov, Anna Ilinskaya, Maria Poptsova, Valery Ilinsky, Alexander Rakitko

**Affiliations:** 1 National Research University Higher School of Economics, Russian Federation, Moscow, Russia; 2 Genotek Ltd., Moscow, Russia; 3 Moscow Center for Advanced Studies, Moscow, Russia; 4 Genotek Center: AI in Personalized Medicine, ITMO University, Saint-Petersburg, Russia; 5 ITMO University, Saint-Petersburg, Russia; 6 Eligens SIA, Riga, Latvia

**Keywords:** hypertension, inner Eurasia, multiethnic populations, precision medicine, PRS

## Abstract

**Introduction:**

Arterial hypertension is one of the leading contributors to cardiovascular morbidity and mortality worldwide. This study aimed to evaluate the performance of polygenic risk scores (PRS) for hypertension in Russia and to develop predictive models integrating PRS and questionnaire-based risk factors for disease risk assessment.

**Methods:**

We analyzed a cohort of 175,704 individuals from multiethnic inner Eurasian populations. Published PRS for systolic blood pressure, diastolic blood pressure, and pulse pressure were evaluated for association with hypertension across different ancestry groups. Predictive models integrating PRS and questionnaire-derived risk factors were developed using multiple machine learning tools, including neural networks.

**Results:**

PRSs for systolic and diastolic blood pressure showed marked differences between the top and bottom deciles of the PRS distribution, with odds ratios of 6.20 (95% CI: 5.22–7.36) and 6.71 (95% CI: 5.58–8.06), respectively. The PRS for pulse pressure was also strongly associated with hypertension, with an odds ratio of 3.71 (95% CI: 3.16–4.35). All evaluated PRSs were consistently associated with hypertension across several ancestry groups represented in Russia and neighboring regions, including East Slavic populations (Russians, Belarusians, and Ukrainians), populations of the Volga–Ural region, such as Tatars, and West Asian–related groups represented by Armenians and Hemshins. A neural network model integrating PRSs with questionnaire-based risk factors achieved a test ROC-AUC of 0.8245 (95% CI: 0.8114–0.8362), demonstrating robust discriminatory performance for arterial hypertension.

**Conclusion:**

Our study demonstrates that previously published PRSs for systolic blood pressure, diastolic blood pressure, and pulse pressure retain substantial predictive value across diverse inner Eurasian populations and provide complementary information beyond conventional questionnaire-based risk factors. Among the evaluated scores, the systolic blood pressure PRS showed the most robust and consistent transferability, remaining informative even in several genetically diverse and underpowered cohorts.

## 1 Introduction

Arterial hypertension (AH) is a major modifiable risk factor for cardiovascular disease (1), stroke (1–3), and chronic kidney disease (4), contributing significantly to global morbidity and mortality (5, 6). The global prevalence of hypertension continues to increase (5), while less than 50% of women and less than 40% of men receive treatment (7). A key determinant of treatment uptake is disease awareness (8), which is traditionally achieved through increased coverage of medical examinations and public information.

While screenings require significant time from healthcare providers and patients, the use of extensive questionnaire data enables the development of predictive models for disease presence. The classification results of these models enable the identification of individuals at high risk of hypertension and recommend a visit to the appropriate doctor. Importantly, such analysis allows for a one-time data collection, minimizing patient and staff involvement. This approach makes early diagnosis more accessible, which plays a significant role in preventing disease progression. Long-term cohort studies have demonstrated that early identification and intervention can prevent or significantly delay these outcomes through lifestyle modification, pharmacological treatment, and personalized risk management strategies (5).

Hypertension is not a static trait, but a dynamic process shaped by both genetic variants and physiological regulation. At the molecular level, blood pressure is maintained through the interplay of neurohumoral, renal, vascular, and central nervous system pathways (9). Key mechanisms include the renin–angiotensin–aldosterone system, sodium handling via epithelial sodium channels, and central sympathetic regulation, all of which can create self-sustaining loops of elevated blood pressure, vascular remodeling, and endothelial dysfunction. Genetic factors contribute substantially to this process, with heritability of blood pressure traits estimated at 30%–50% (10). Large-scale genome-wide association studies (GWAS) have revealed a highly polygenic architecture involving hundreds of common variants with modest effects (11–16). These discoveries led to the development of PRSs, which integrate the cumulative impact of multiple alleles (17, 18) to stratify individuals by their inherited predisposition to hypertension (19–29). The increased availability of sequencing makes it possible to include PRS statistics in prediction models, along with questionnaire data (24, 30).

The problem of controlling AH remains unresolved in many countries, including Russia (8). This territory is characterized by distinctive environmental exposures, including high dietary sodium intake, elevated alcohol consumption, smoking prevalence, psychosocial stress, and variable healthcare access compared with Western Europe. The circumstances described above raise questions about the transferability of results obtained in Europe and the USA (31) to Russia.

To date, PRS have been predominantly developed and validated in populations of European descent, raising concerns about their applicability across ancestries (19, 31–33). As a result, there is growing interest in developing and validating PRSs in non-European and admixed populations, including those from Russia (34–37), as well as assessing the performance of existing scores (19, 32, 38–40). Russia is a multinational country in inner Eurasia (41) that encompasses numerous populations with diverse ancestral backgrounds. The association between PRS values and the occurrence of hypertension was evaluated across many of these groups.

Thus, in our study, we analyzed a cohort of 175,704 individuals from present-day inner Eurasian populations (41). Based on questionnaire data and calculated PRS values, we constructed predictive models for hypertension. The PRSs used were also validated for different ethnic groups (42).

## 2 Materials and methods

### 2.1 Study cohort

We analyzed the genetic data of 175,704 individuals from the database of Genotek, the consumer genetics and research company (45). All participants provided informed consent for their data to be used for research purposes and took an online questionnaire.

### 2.2 Phenotype and risk factors construction

AH phenotype was defined based on self-reported medical history collected through a structured questionnaire. Participants were presented with a list of diseases corresponding to ICD-10 codes, and diagnoses were selected from predefined drop-down options to ensure standardized reporting. Individuals were classified as hypertensive if they reported any diagnosis of essential (primary) hypertension (I10), hypertensive heart disease with predominant heart involvement (I11), hypertensive heart disease with predominant kidney involvement and renal insufficiency without signs of hypertensive crisis (I12), hypertensive heart disease with both heart and kidney involvement (I13), or secondary hypertension (I15). All hypertension subtypes were treated as a single unified phenotype (arterial hypertension, AH), and no further subclassification was considered in downstream analyses; therefore, potential misclassification between hypertension subtypes is not expected to affect the results. In addition, participants who did not report any hypertension diagnosis but indicated current use of antihypertensive medications (including metoprolol, captopril, and carvedilol) were also classified as having AH. This approach is consistent with definitions used in large-scale biobank studies, including studies based on self-reported data in population-scale resources such as the UK biobank project (43).

The following risk factors were considered in the predictive models: age, sex, body mass index (BMI), smoking status (current, former, or never), alcohol consumption (never, monthly or less, 2–4 times per month, 2–3 times per week, 4 or more times per week), education level (preschool, school, undergraduate university degree, specialist or MA/MSc, PhD), work (unemployed, sedentary, moderately active, highly active), daily step count (<5,000, 5,000–10,000, ≥10,000), and physical training.

To improve the robustness of downstream analyses, participants with extreme values of age and body mass index (BMI) were excluded before the analysis. Individuals younger than 20 years or older than 80 years were removed to restrict the analysis to the adult population while excluding sparsely represented age groups at the distribution tails (Supplementary Figures S1A,B). Participants with BMI values below 18 kg/m^2^ or above 40 kg/m^2^ were also excluded. These thresholds approximately correspond to the boundaries of underweight and class III (morbid) obesity according to the World Health Organization BMI classification system (44).

### 2.3 Genotyping, quality control, and imputation

DNA extraction and genotyping were performed on saliva samples. Out of 175,704 samples analyzed, 120,563 were genotyped using the Illumina Infinium Global Screening Array v.3 microarrays (∼650,000 SNPs). All samples were processed in batches of 192–768 samples. The GenomeStudio software (Illumina, San Diego, CA) and manually created cluster files were used to cluster the raw signals and call the genotypes. SNPs with a call rate <0.9 and individuals with a sample call rate <0.97 within the batch were removed. Then, genotype imputation was performed using HRC and 1000 Genomes reference panels with Beagle 5.1 (45). Imputed variants with DR2 > 0.7 were kept for the downstream analysis.

Out of 175,704 samples analyzed, 55,141 were sequenced using a variable-depth whole-genome sequencing (vdWGS) approach. Libraries were prepared using a PCR-based protocol with additional targeted enrichment of regions of interest via custom hybridization probes. Sequencing was performed on an Illumina NovaSeq 6000 platform using 150 bp paired-end reads. Samples were sequenced in batches of 768, achieving low genome-wide coverage (∼1x) supplemented by higher coverage (∼30x) in targeted regions, specifically the exons of genes included in the custom panel. Paired-end sequencing reads were aligned to the reference genome (hg19) using BWA-MEM2 (v. 2.2.1) (46) (alignment score ≥ 30) with duplicate reads marked. The resulting SAM stream was directly piped to GATK (v. 4.2.0) (47) SortSam tool to convert and sort the output by coordinate, producing a sorted BAM file. Samples with fewer than 5,000,000 mapped reads or contamination level exceeding 0.1 were excluded from further analysis (48). Aligned reads were used to further call target regions using GATK HaplotypeCaller to generate genomic VCF (gVCF) files with a minimum base quality score of 20 and a minimum mapping quality of 10. gVCF files were filtered using bcftools view (49) to retain only sites with a read depth of at least 10. Genotypes were then converted to VCF with GATK GenotypeGVCFs. Imputation of non-targeted regions was performed from BAM-derived genotype likelihoods using GLIMPSE2 (v2.0.0) (50) against a modified Haplotype Reference Consortium (HRC) reference panel (r1.1) (51) that was enriched with variant positions unique to the 1000 Genomes Project (Phase3 v5) (52), while preserving all original HRC positions unchanged. The resulting called and imputed genotypes were merged into a single VCF file. Imputed genotypes with a maximum genotype probability below 0.95 were filtered out, and variants with a raw allele frequency (RAF) below 0.001 were also excluded.

### 2.4 PRS validation

Polygenic risk scores (PRSs) for systolic blood pressure (SBP), diastolic blood pressure (DBP), and pulse pressure (PP) were calculated for each participant using previously published PRS data (19). PRS data were downloaded from the PGS Catalog website (https://www.pgscatalog.org) using accession codes PGS004603, PGS004604, and PGS004605, hereinafter referred to as PRS_SBP, PRS_DBP, and PRS_PP, respectively. The original SNP effect sizes provided in the published PGS models were applied directly to our genotype data to calculate individual PRS values. The underlying GWAS summary statistics were derived from a meta-analysis of up to 1,028,980 individuals and 7,584,058 SNPs. Genome-wide significant loci were defined using a threshold of *P* < 5 × 10^−8^, with linkage disequilibrium (LD) pruning (*r*^2^ < 0.1 within a 1 Mb window). Known loci and correlated variants (LD *r*^2^ > 0.1 within ±500 kb) were excluded before the clumping and LD-pruning to identify independent loci. Additional details, including the number of genome-wide significant variants for each trait, are provided in Supplementary Table S1 and can be found in the original publication (19). The corresponding variant lists and GWAS summary statistics are not reproduced here but are accessible via the GWAS Catalog using accession codes GCST90310294, GCST90310295, and GCST90310296.

To validate association with hypertension, each PRS was partitioned into deciles (1st–10th) using quantile-based binning. Odds ratios (ORs) and 95% confidence intervals (CIs) were estimated using logistic regression models comparing each PRS decile with the lowest decile, adjusted for age and sex. Separate models were fit for each decile comparison. ORs were obtained by exponentiating regression coefficients, and CIs were derived using the Wald method (*β* ± 1.96 × SE). Results were visualized as ORs across deciles with 95% CI bands and a reference line at OR = 1.

### 2.5 Combined model for AH prediction

Categorical features (smoking status, alcohol consumption, education, steps, work, training, sex) were converted to numerical form using one-hot encoding for neural networks and ordinal encoding for other models. All numerical and continuous features (age, BMI) were standardized using *z*-score normalization. Engineered variables included age × BMI, PRS_DBP × PRS_SBP, PRS_DBP × PRS_PP, and PRS_SBP × PRS_PP (polygenic score interactions), as well as bad habits (a composite of smoking and excessive alcohol consumption), which were also standardized. The dataset was split 80/20 into training and test sets with stratification based on the presence of hypertension.

Feature selection combined Information Value (IV) analysis and Weight of Evidence (WOE) encoding of binned numerical features (age, BMI, PRS_SBP, PRS_DBP, PRS_PP, age × BMI, PRS_DBP × PRS_SBP, PRS_DBP × PRS_PP, PRS_SBP × PRS_PP), along with WOE encoding of categorical features. This was followed by model-specific Sequential Feature Selection.

The selected features were used to train and evaluate multiple machine learning algorithms: Logistic Regression, Random Forest, CatBoost, LightGBM, XGBoost, a TensorFlow-based neural network model (Dense NN), and a ResNet-like neural network architecture. Hyperparameters for each model were optimized via Optuna (100–1,000 trials) to maximize ROC-AUC while minimizing the train–test performance gap. Performance was evaluated using ROC-AUC, along with Accuracy, Precision, Recall, and *F*1-score. The best-performing model was interpreted using SHAP analysis.

In addition, the best model, which includes both PRSs and questionnaire-based characteristics (age, smoking status, alcohol consumption, education, work, training, sex, BMI, daily steps), was compared with the basic model based only on PRS and with the model based solely on the characteristics obtained from the questionnaire, using the DeLong test for statistical evaluation.

### 2.6 Definition of ancestry

Genetic ancestry for each individual was inferred using an in-house local ancestry inference algorithm that has been applied in prior studies (37, 53, 54). Briefly, the genome was divided into windows, and within each window, the Positional Burrows–Wheeler Transform (PBWT) algorithm was applied to identify population-specific haplotype matches. The resulting PBWT features were aggregated and classified with a Random Forest model, followed by a custom Hidden Markov Model that corrected phasing and classification errors. This approach was trained on a reference panel of 17,559 individuals of known ancestry, representing 101 distinct populations, which were subsequently clustered into 22 broader population groups based on genetic similarity. The panel aggregates previously published genomic data (52, 55–58) with genotype data from Genotek clients. For the latter, individuals were included only if genetic ancestry assignment was consistent with a single inferred population cluster across three generations of reported relatives, based on available genealogical and genetic information. Classification performance across the population groups demonstrated high overall accuracy on the people with known ancestry (Supplementary Figure S2). For each study participant, ancestry proportions across these groups were estimated from local ancestry inference. Individuals were assigned to a population group when the dominant ancestry fraction exceeded 70%.

### 2.7 Validation in different genetic ancestry groups

To evaluate the strongest associations with AH across diverse ancestry groups, we defined subpopulations using an in-house ancestry inference algorithm (see Section 2.6). Subgroup analyses were restricted to populations with enough hypertension cases to avoid sparse-data bias and unstable effect estimation in logistic regression models. Groups with fewer than 10 cases or exhibiting complete separation of outcome by sex were excluded from subgroup-specific analyses. For each eligible ancestry group, we applied a generalized linear model to test the association between PRSs and AH, including age and sex as covariates. The *p*-values obtained across subgroup analyses were adjusted for multiple testing using the Benjamini–Hochberg false discovery rate correction.

## 3 Results

### 3.1 Study cohort description

Our analysis included a substantial cohort of 175,704 individuals, with women accounting for 54.6% of the sample. The median age was 37 years [interquartile range (IQR): 30–45], and the median BMI was 24.07 kg/m^2^ (IQR: 21.30–27.34). AH was reported in 4,147 individuals (2.4%), with the presence of several diagnoses from those considered (I10–I15) in a small number of participants (Supplementary Figure S3). For a more detailed overview of the cohort's demographic and clinical characteristics, refer to Supplementary Table S2.

In the study population, categorical trait distributions differed substantially between individuals with AH and controls (Supplementary Figures S4A,B). The proportion of males was higher among individuals with AH. Distributions of smoking and alcohol consumption were broadly similar between groups, with only modest differences across categories. Individuals with AH were slightly more represented in lower physical activity and training frequency categories. Educational level and work status showed minor differences between groups without clear shifts in a single category.

For quantitative traits, individuals with AH were older and had higher BMI compared to controls. Age and BMI distributions were right-skewed, with higher central tendencies in the AH group (Supplementary Figures S5A–C).

### 3.2 PRS validation

**Figure 1:**
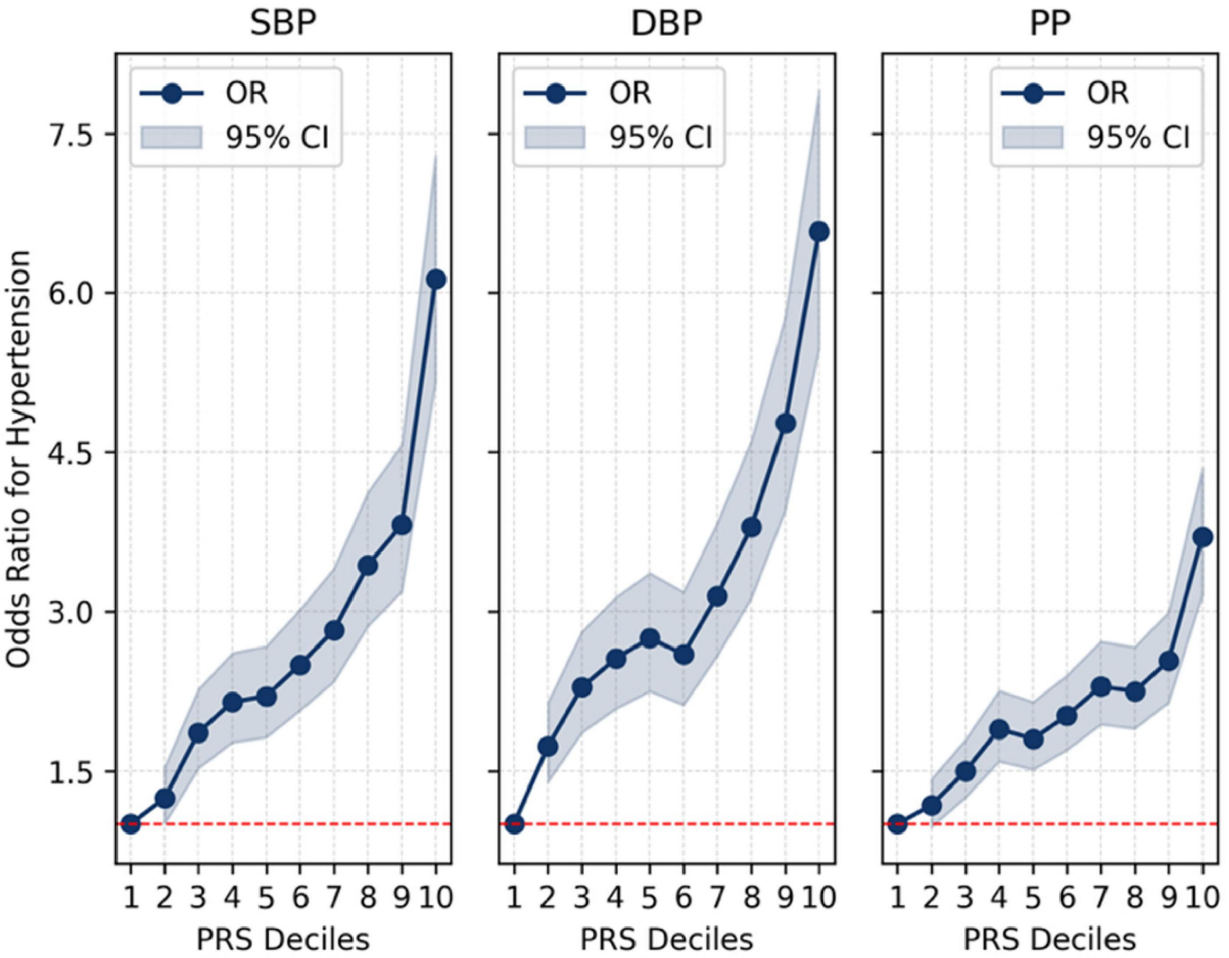
Association between PRSs and hypertension. ORs and 95% confidence intervals (CIs) for hypertension are shown across deciles of PRSs for diastolic blood pressure (DBP PRS), systolic blood pressure (SBP PRS), and pulse pressure (PP PRS). Individuals in the top decile of DBP and SBP PRSs had a more than 6-fold increased risk of hypertension compared with those in the lowest decile, whereas the PP PRS showed a more modest but significant effect.

While lifestyle and environmental determinants are critical, recent advances in PRSs underscore the role of inherited predisposition. We observed significant positive associations between three PRSs: PRS_SBP, PRS_DBP, and PRS_PP, and hypertension prevalence after adjustment for age and sex (Figure 1, Supplementary Table S3). The strongest effects were seen for PRS_SBP and PRS_DBP, with individuals in the highest decile showing a more than six-fold increase in hypertension risk compared with the lowest decile (PRS_SBP: OR = 6.20, 95% CI 5.22–7.36; PRS_DBP: OR = 6.71, 95% CI 5.58–8.06). PRS_PP also demonstrated a robust, though attenuated, association, with the top decile conferring a nearly 4-fold higher risk (OR = 3.71, 95% CI 3.16–4.35). These findings highlight the potential utility of PRSs for stratifying hypertension risk and guiding early prevention strategies, including in inner Eurasian populations.

### 3.3 Machine learning models for hypertension prediction

The results of feature selection based on IV analysis, classifying predictors according to their discriminatory ability in relation to AH, are presented in Supplementary Table S4. Age and BMI-related variables showed very strong predictive power, polygenic scores demonstrated a medium value, while lifestyle and sociodemographic factors contributed weak discrimination. Smoking status, steps, and bad habits were excluded due to very weak predictive performance.

Among all models, the Dense NN achieved the highest ROC-AUC (0.8245 [95% CI: 0.8118–0.8362]). The model consisted of an input layer followed by two hidden dense layers with 128 and 48 neurons, respectively. Each hidden layer used the ReLU activation function and dropout regularization with a dropout rate of 0.18. The output layer contained a single neuron with a sigmoid activation function to model the binary outcome. The model was trained using the SGD optimizer with a learning rate of 0.047 and binary cross-entropy as the loss function.

The Dense NN was followed closely by ResNet (ROC-AUC = 0.8231 [95% CI: 0.8118–0.8362]). Classical machine learning models demonstrated slightly lower but comparable performance, including Logistic Regression (ROC-AUC = 0.7982 [95% CI: 0.7843–0.8116]), CatBoost (ROC-AUC = 0.7979 [95% CI: 0.7840–0.8119]), XGBoost (ROC-AUC = 0.7977 [95% CI: 0.7839–0.8119]), and LightGBM (ROC-AUC = 0.7971 [95% CI: 0.7828–0.8109]) (Table 1, Supplementary Table S5).

**Table 1:** Model performance and hyperparameters.

Model	ROC AUC (95% CI)	PR AUC	MCC	Precision	Recall	Specificity	Balanced accuracy	Brier score
Dense NN	0.8245 (0.8118–0.8362)	0.1263	0.2021	0.1016	0.6092	0.8500	0.7296	0.0248
ResNet	0.8231 (0.8102–0.8353)	0.1280	0.2042	0.1670	0.3277	0.9545	0.6411	0.0248
Logistic regression	0.7982 (0.7843–0.8116)	0.1120	0.1883	0.1014	0.5420	0.8662	0.7041	0.1284
CatBoost	0.7979 (0.7840–0.8119)	0.1152	0.1891	0.1122	0.4769	0.8949	0.6859	0.0251
XGBoost	0.7977 (0.7839–0.8116)	0.1180	0.1881	0.0953	0.5872	0.8448	0.7160	0.0251
LightGBM	0.7971 (0.7828–0.8109)	0.1167	0.1887	0.0937	0.6040	0.8373	0.7206	0.0251
Dense NN (questionnaire features)	0.7913 (0.7777–0.8048)	0.0975	0.1758	0.0980	0.5042	0.8708	0.6875	0.0254
Random forest	0.7872 (0.7723–0.8016)	0.1153	0.1894	0.1214	0.4307	0.9132	0.6719	0.0252
Dense NN (PRS only)	0.6404 (0.6211–0.6577)	0.0496	0.0718	0.0550	0.3015	0.8557	0.5786	0.0262

**Figure 2:**
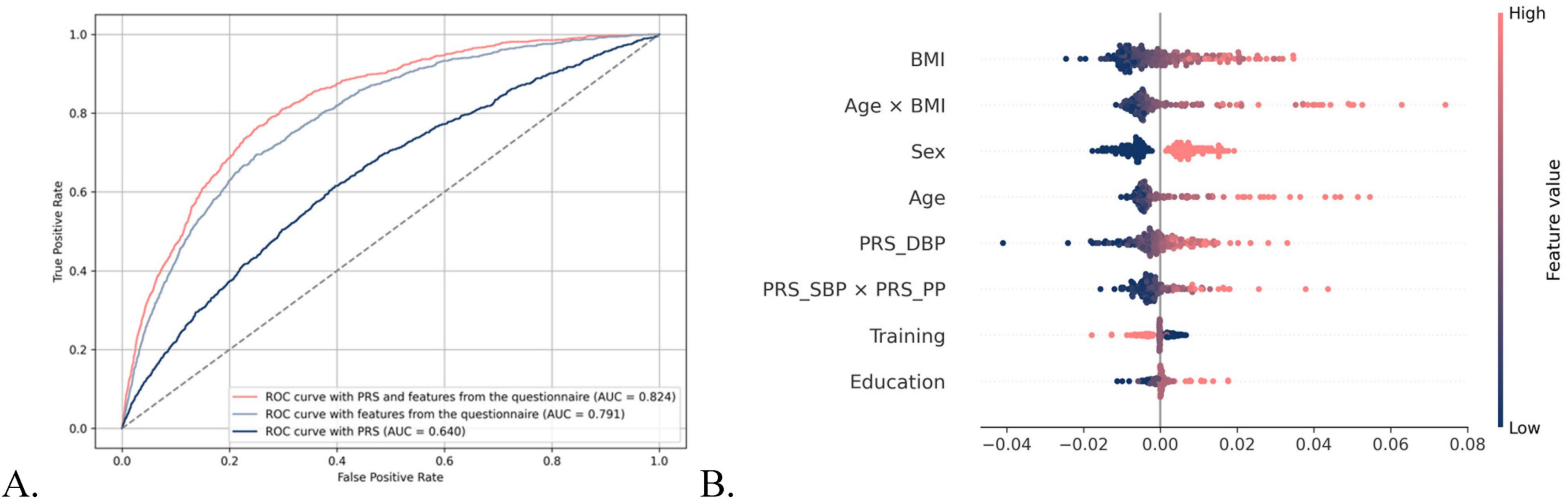
Performance and feature importance of hypertension prediction models **(A)** ROC curves comparing models based on PRS (AUC = 0.640), questionnaire-derived features (AUC = 0.791), and a combined model integrating both sources of information (AUC = 0.824). The integration of PRS with questionnaire data improved predictive performance. **(B)** SHAP summary plot for the combined model. The most influential features were BMI, age × BMI, and sex, with additional contributions from polygenic scores (PRS_DBP, PRS_SBP × PRS_PP), as well as age, training and education.

Models trained only on questionnaire-based features demonstrated slightly reduced but still robust discrimination (Dense NN with questionnaire features: ROC-AUC = 0.7931 [95% CI: 0.7795–0.8060]). In contrast, the neural network trained using only PRSs showed substantially lower predictive performance (ROC-AUC = 0.6404 [95% CI: 0.6211–0.6577]), underscoring the limited standalone predictive capacity of genetic scores (Figure 2A).

SHAP analysis of the best-performing neural network model, which incorporated all features, revealed that the BMI exerted the strongest influence on hypertension prediction, followed by the combined variable age × BMI and sex (Figure 2B). Higher values of these features were consistently associated with an increased model output. Among polygenic predictors, PRS_DBP and PRS_SBP × PRS_PP contributed substantially, while age, training, and education also demonstrated notable effects (see Supplementary Table S6 for SHAP values of feature importance).

### 3.4 PRS analyses in inner Eurasian ancestry

**Figure 3:**
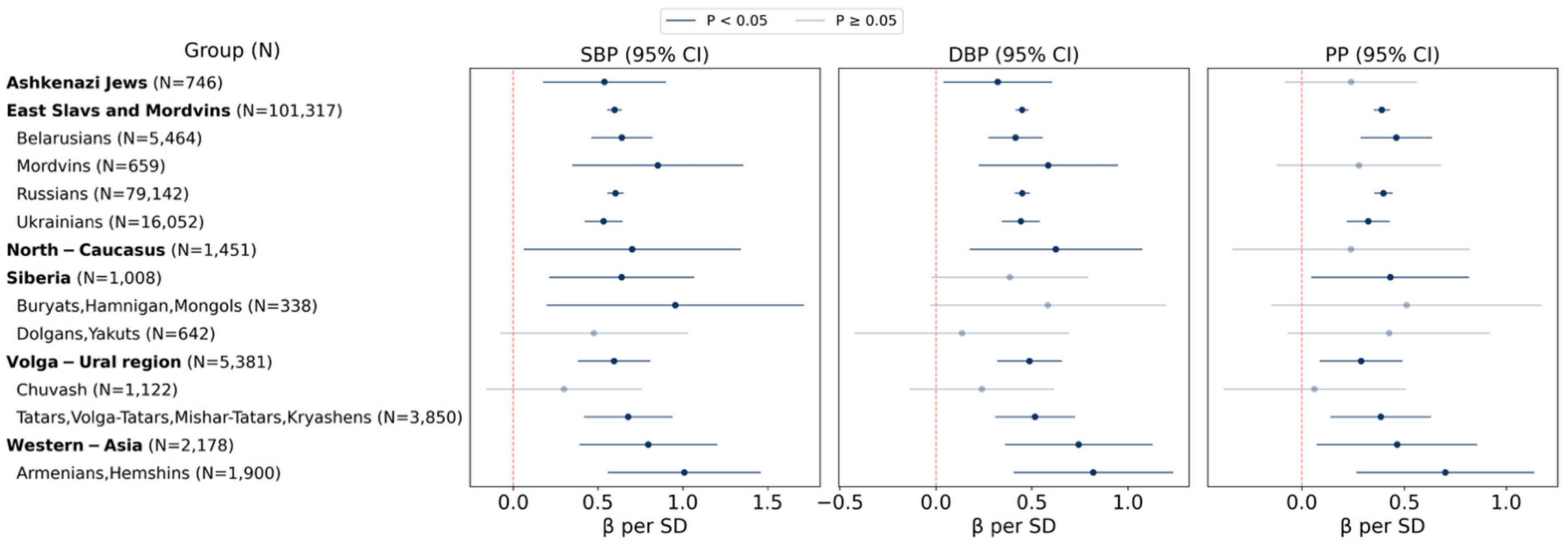
Forest plots show β-estimates and 95% confidence intervals for the associations between three polygenic risk scores (PRS_SBP, PRS_DBP, and PRS_PP) and blood pressure traits, including systolic blood pressure (SBP), diastolic blood pressure (DBP), and pulse pressure (PP). Analyses were performed separately for each ancestry group.

The transferability of previously published PRSs for SBP, DBP, and PP (19) varied substantially across the populations of inner Eurasia within our cohort (Figure 3, Supplementary Table S7). The strongest and most consistent associations were observed in the large East Slavs and Mordvins meta-group (*N* = 101,317), where all three PRSs were significantly associated with hypertension-related traits after multiple testing correction. Similar robust effects were observed in Russians, Ukrainians, and Belarusians analyzed separately, indicating stable PRS performance across East Slavic populations.

Among the Volga–Ural populations, significant associations for all three PRSs were detected in the combined regional cohort and were primarily driven by Tatars, where DBP-, SBP-, and PP-based PRSs all showed significant effects. In contrast, no significant associations were observed in the Chuvash cohort, which is characterized by a relatively small sample size and sex imbalance, including only 27 individuals with hypertension, 9 of whom were female. Western Asian populations demonstrated particularly strong effect sizes despite relatively small numbers of hypertension cases. In Armenians and Hemshins (*N* = 1,900; 28 cases, 10 females), all three PRSs remained significant after multiple testing correction, with the SBP PRS showing the largest observed effect size. Similar results were obtained for the broader Western Asia cluster.

Several smaller cohorts also showed partial but notable PRS performance. In the Siberian populations, significant associations were detected for SBP- and PP-based PRSs despite the low number of cases (37 cases, 20 females), whereas the DBP PRS showed only nominal significance. Likewise, in Buryats, Hamnigans, and Mongols, the SBP PRS remained significant despite the very limited sample size (14 cases, 9 females), whereas DBP and PP PRSs did not survive multiple-testing correction. Conversely, some cohorts with larger sample sizes still demonstrated limited predictive performance. For example, the North Caucasus group showed significant associations for DBP and SBP PRSs but not for PP, whereas Dolgans and Yakuts exhibited no significant associations despite sample sizes comparable to several successfully replicated cohorts.

Overall, the SBP PRS demonstrated the most consistent transferability across populations, remaining significant even in several underpowered cohorts. The DBP PRS showed moderately stable performance but appeared more sensitive to sample size and population structure. In contrast, the PP PRS displayed the weakest and least consistent replication pattern.

## 4 Discussion

Our study provides one of the first large-scale validations of PRSs for SBP, DBP, and PP in a cohort representing populations of inner Eurasia. Consistent with prior epidemiological findings (5, 59, 60), we confirmed well-established associations of hypertension with age, BMI, and sex, whereas lifestyle and sociodemographic factors showed limited discriminative value; smoking status, step count, and other behavioral variables made an even weaker contribution.

Importantly, PRSs provided an additional layer of risk stratification beyond questionnaire-based predictors. While models trained exclusively on questionnaire-derived features achieved only slightly reduced discrimination (ROC-AUC = 0.7931 [95% CI: 0.7795–0.8060]) compared to the best-performing model incorporating PRSs (Dense NN: ROC-AUC = 0.8245 [95% CI: 0.8118–0.8362]), the observed improvement suggests that genetic information contributes a complementary signal beyond conventional risk factors. In contrast, a model trained using PRSs alone demonstrated substantially lower predictive performance (ROC-AUC = 0.6404 [95% CI: 0.6211–0.6577]), underscoring the limited standalone utility of genetic scores.

Beyond ROC-AUC of 0.8245 (95% CI: 0.8118–0.8362), the best model showed consistent performance across complementary metrics, including a PR AUC of 0.1263, reflecting a meaningful predictive signal under strong class imbalance, and a Matthews correlation coefficient (MCC) of 0.2021, indicating modest but stable overall classification quality across all confusion matrix components. The balanced accuracy (0.7296) further supports consistent performance across sensitivity and specificity, while the Brier score (0.0248) suggests well-calibrated predicted probabilities. A detailed comparison of model characteristics and performance is presented in Supplementary Table S5.

These results are broadly comparable to, though slightly lower than, the performance reported by Cheraghi et al., who obtained an AUC of 0.87 (95% CI: 0.786–0.954) in a substantially smaller cohort of 303 adults (61). This suggests that model performance may benefit from smaller, more homogeneous samples but risks overfitting. In contrast, Gideon MacCarthy et al. reported a lower predictive accuracy with an AUC of 0.71 in a large-scale cohort of 244,718 Europeans, highlighting the challenges of hypertension prediction in highly heterogeneous population-based datasets (62). Meanwhile, a recent Japanese study by Tanaka et al., conducted in 15,965 participants, achieved an AUC of 0.825 (95% CI: 0.802–0.839), closely aligning with our findings despite differences in ancestry and cohort size (63). Collectively, these comparisons underscore the consistency of our model's predictive validity across populations and demonstrate its competitive performance relative to prior studies.

The most robust and reproducible transferability of previously published PRSs was observed in Russians, Ukrainians, and Belarusians, where all three PRSs remained highly significant after multiple-testing correction. Although the transferability of these PRSs was not confirmed in some inner Eurasian populations, we suggest that this is primarily attributable to limited sample sizes in several subgroups rather than genuinely poor PRS performance. This assumption is also supported by the fact that the scores were previously successfully validated on more distant African-American ancestry populations (19) and were discussed in other works (39, 64, 65).

At the same time, it should be noted that some cohorts of comparable size demonstrated different statistical outcomes. For example, Armenians and Hemshins showed significant associations for all three PRSs despite including only 28 hypertension cases (10 females), while Buryats, Hamnigans, and Mongols retained significant SBP PRS associations with only 14 cases (9 females). In contrast, the Chuvash cohort, which included 27 cases, and the Dolgans and Yakuts, with 22 cases, failed to demonstrate significant associations for any of the PRSs.

Among the evaluated scores, the SBP PRS demonstrated the strongest and most consistent transferability, remaining significant even in several underpowered cohorts and across genetically diverse populations. In contrast, the DBP PRS showed somewhat lower robustness, whereas the PP PRS displayed the least stable replication pattern.

Despite these strengths of our analysis, several limitations should be considered. First, hypertension status was self-reported rather than clinically ascertained, and the precise age of onset was unavailable. While questionnaire-based features provide scalable and low-cost data (66), their reliance on self-report may limit precision in clinical translation.

Taken together, our findings demonstrate both the promise and the current limitations of PRS-based prediction in diverse populations. They provide evidence that PRSs are informative for hypertension risk in inner Eurasia, while also revealing ancestry-specific differences that caution against one-size-fits-all implementation. Future work should focus on longitudinal validation, incorporation of clinical endpoints, and the development of ancestry-aware or locally trained PRSs that capture region-specific genetic and environmental architectures.

## Data Availability

The data analyzed in this study is subject to the following licenses/restrictions: Genotek’s data cannot be accessed as it is the client's private information. Requests to access these datasets should be directed to info@genotek.ru.
