# Supplementary material for: Machine learning prediction of hypertension integrating polygenic risk scores in inner Eurasian populations
Source: Front Cardiovasc Med. 2026 Jul 8;13:1843103. doi: 10.3389/fcvm.2026.1843103 (PMC13417086; doi:10.3389/fcvm.2026.1843103)
Supplement: Supplementary file 1 [file Supplementaryfile1.pdf]

Supplementary Material

1 Supplementary Data

2 Supplementary Figures and Tables

2.1 Supplementary Figures

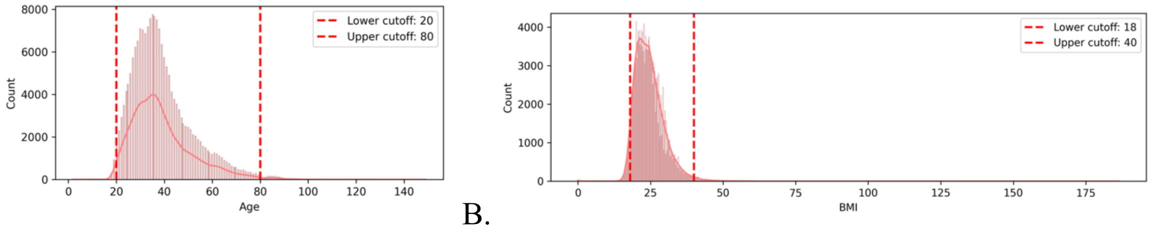

A. B.

Supplementary Figure 1. Empirical distributions of participant age (A) and body mass index (BMI) (B) used for sample quality control. Red dashed lines indicate the lower and upper exclusion thresholds applied in downstream analyses.

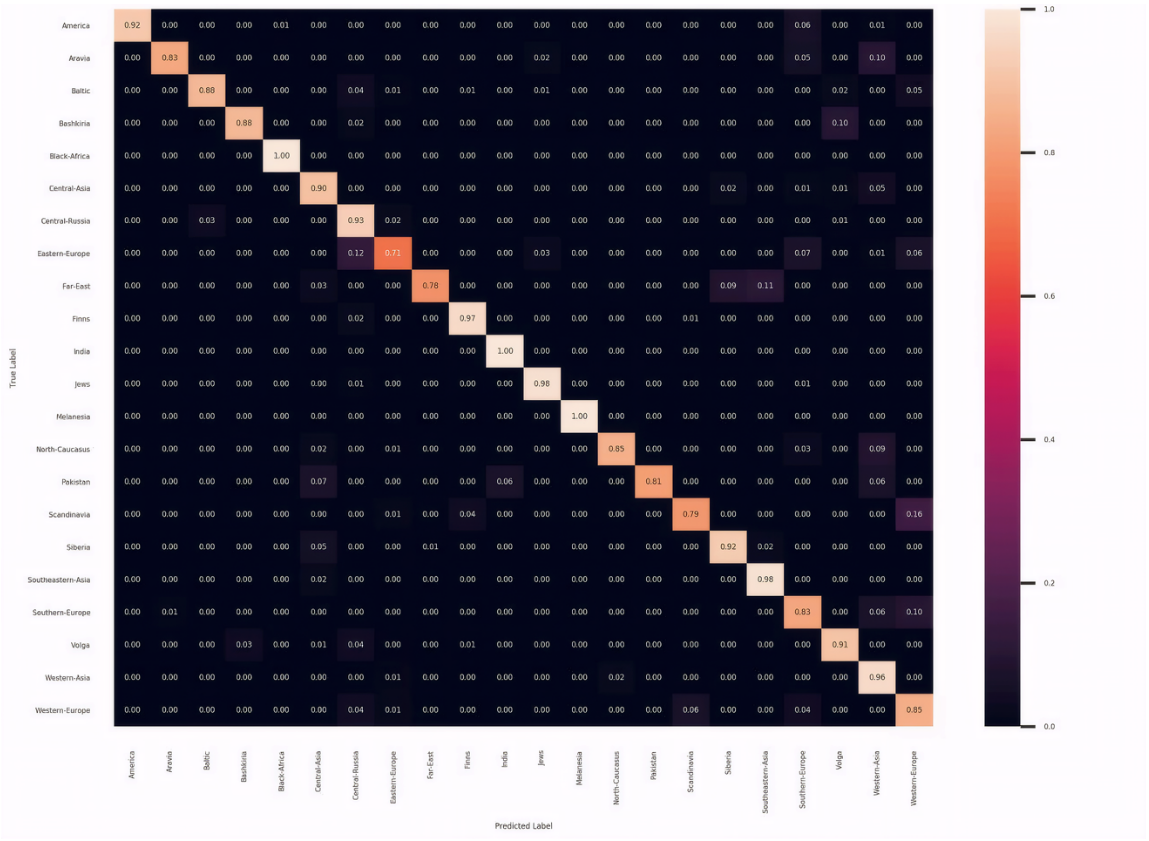

Supplementary Figure 2. Normalized confusion matrix for ancestry classification

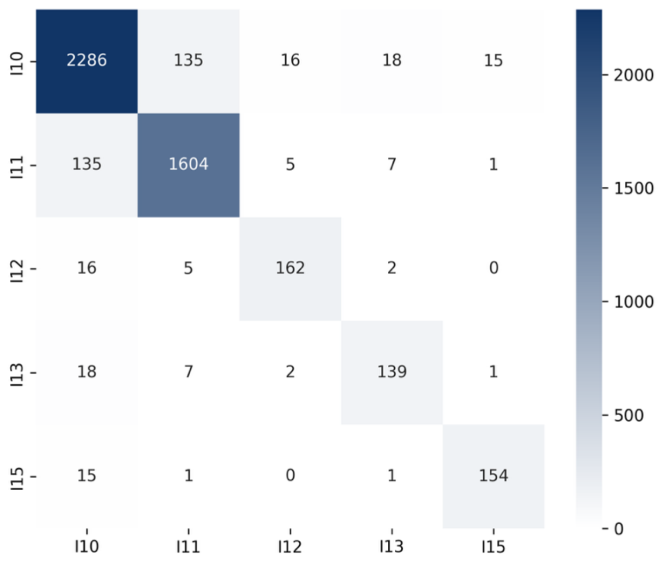

Supplementary Figure 3. ICD-10 Diagnosis overlap

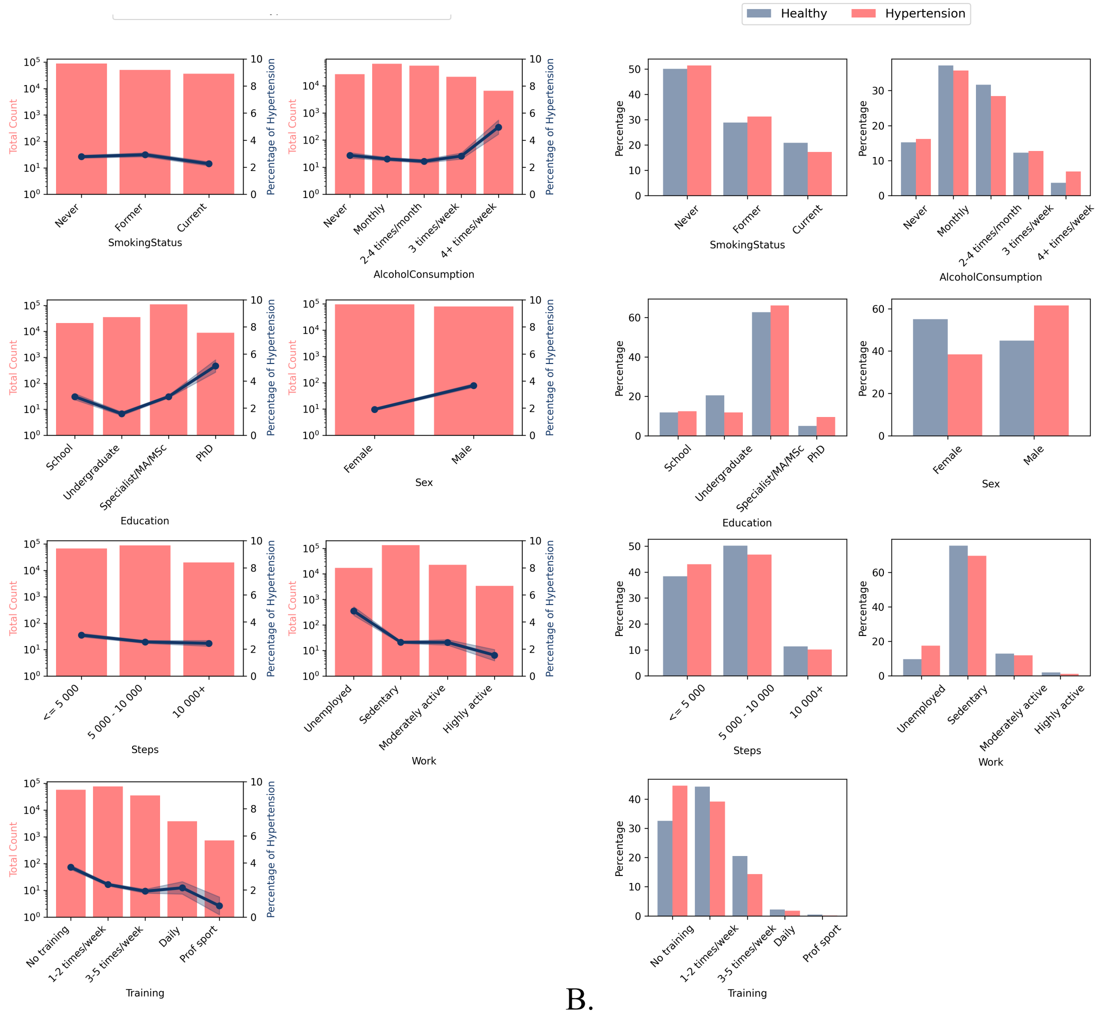

A. B.

Supplementary Figure 4. Distribution of categorical characteristics in relation to arterial hypertension in a Russian cohort. **(A)** Total counts of participants across categories with the corresponding percentage of hypertension indicated by the line. **(B)** Percentage distribution of participants with and without hypertension across the same categories.

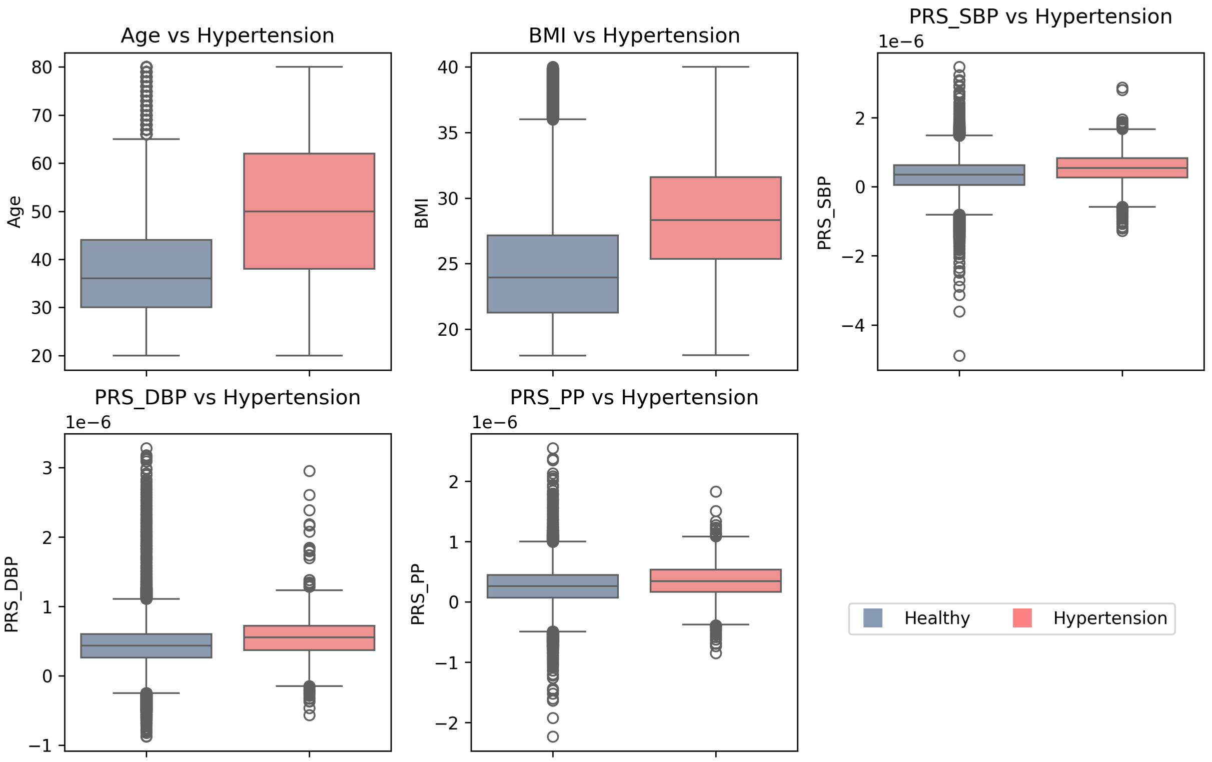

A.

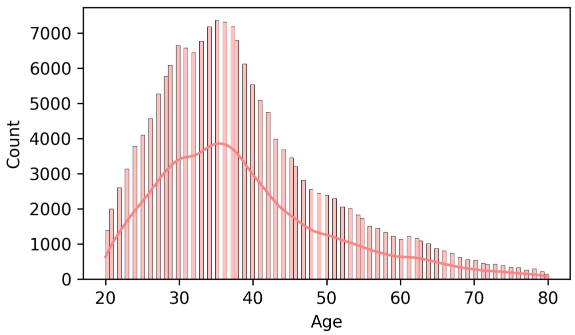

B.

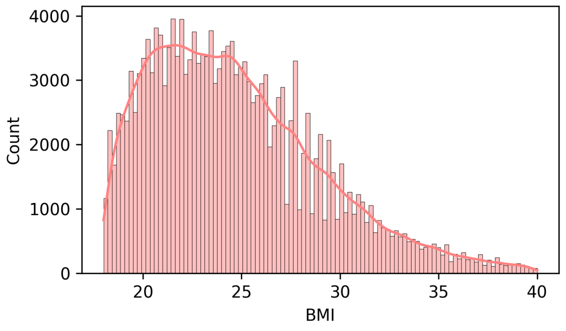

C.

Supplementary Figure 5. Distribution of quantitative characteristics depending on the presence of arterial hypertension. (**A**) Boxplots showing the distribution of age, BMI, and polygenic risk scores (PRS for SBP, DBP, and PP) in individuals with and without hypertension. **(B)** Distribution of age in the cohort. **(C)** Distribution of BMI in the cohort.

2.2 Supplementary Tables

Supplementary Table 1. Overview of PGS Catalog scores used in the study.

Trait	PGS Catalog data accession code	GWAS Catalog data accession code	Number of GWAS-level significant variants	P-value threshold	LD pruning criteria	GWAS sample size
Systolic blood pressure (SBP)	PGS004603	GCST90310294	1,495 loci	$P < 5 \times 10^{-8}$	$r^2 < 0.1$ within 1 Mb; known loci excluded ($r^2 > 0.1$ within ±500 kb); clumping and LD-pruning applied	up to 1,028,980 individuals
Diastolic blood pressure (DBP)	PGS004604	GCST90310295	1,504 loci	$P < 5 \times 10^{-8}$	$r^2 < 0.1$ within 1 Mb; known loci excluded ($r^2 > 0.1$ within ±500 kb); clumping and LD-pruning applied	up to 1,028,980 individuals
Pulse pressure (PP)	PGS004605	GCST90310296	1,318 loci	$P < 5 \times 10^{-8}$	$r^2 < 0.1$ within 1 Mb; known loci excluded ($r^2 > 0.1$ within ±500 kb); clumping and LD-pruning applied	up to 1,028,980 individuals

Supplementary Table 2. Characteristics of the study population.

* Drug consumption refers to participants receiving antihypertensive therapy, including metoprolol, captopril, or carvedilol. ** Healthy individuals without a diagnosis of hypertension (i.e., normotensive participants), regardless of the presence of other non-hypertensive conditions.

Item	All	Female	Male
**N**	**175 704**	95 950 (54.6%)	79 754 (45.4%)
**Age (mean, sd)**	**38.91, 11.91**	39.09, 12.08	38.69, 11.70
**BMI (mean, sd)**	**24.72, 4.35**	23.59, 4.31	26.08, 3.99
**Education**	**School**	10 128 (10.6%)	10 751 (13.5%)
	**Undergraduate University Degree**	61 655 (64.3%)	48 649 (61.0%)
	**Specialist or MA/MSc**	3 769 (3.9%)	5 111 (6.4%)
	**PhD**	20 398 (21.3%)	15 243 (19.1%)
**Smoking**	**Never**	53 179 (55.4%)	35 054 (44.0%)
	**Former**	25 554 (26.6%)	25 368 (31.8%)
	**Current**	17 217 (17.9%)	19 332 (24.2%)

**Alcohol**	**Never**	14 710 (15.3%)	12 095 (15.2%)
	**Monthly or less**	39 451 (41.1%)	25 786 (32.3%)
	**2-4 times per month**	29 194 (30.4%)	26 331 (33.0%)
	**3 times per week**	10 003 (10.4%)	11 522 (14.4%)
	**4 or more times per week**	2 592 (2.7%)	4 020 (5.0%)
**Training**	**No training**	33 464 (34.9%)	24 299 (30.5%)
	**1-2 times a week of light exercise**	45 087 (47.0%)	32 476 (40.7%)
	**3-5 times a week, moderately heavy workouts**	15 439 (16.1%)	20 362 (25.5%)
	**Daily moderately heavy workouts**	1 690 (1.8%)	2 158 (2.7%)
	**Daily over-intensive workouts (Professional sport)**	270 (0.3%)	459 (0.6%)
**Steps**	**5 000 steps and less (45 minutes)**	38 947 (40.6%)	28 749 (36.0%)
	**5 000 – 10 000 steps (45 minutes – 1.5 hours)**	48 160 (50.2%)	39 921 (50.1%)
	**More than 10 000 steps (over 1.5 hours)**	8 843 (9.2%)	11 084 (13.9%)
**Work**	**Unemployed**	13 710 (14.3%)	3 543 (4.4%)
	**Sedentary**	69 980 (72.9%)	62 400 (78.2%)
	**Moderately active**	10 970 (11.4%)	11 673 (14.6%)
	**Highly active**	1 290 (1.3%)	2 138 (2.7%)
**Hypertension**	**I10**	720 (0.8%)	1 566 (2.0%)
	**I11**	651 (0.7%)	953 (1.2%)
	**I12**	66 (0.1%)	96 (0.1%)
	**I13**	65 (0.1%)	74 (0.1%)
	**I15**	69 (0.1%)	85 (0.1%)

	**Drug consumption***	414 (0.4%)	384 (0.5%)
	**Healthy****	94 119 (98.1%)	76 25 (96.3%)

Supplementary Table 3. ORs and 95% CIs from Logistic Regression Models for PRSs Adjusted for Age and Sex (deciles)

	**PRS SBP**			**PRS DBP**			**PRS PP**		
	OR	2.50%	97.50%	OR	2.50%	97.50%	OR	2.50%	97.50%
Intercept	0	0	0	0	0	0	0	0	0
Decile 2	1.24	1	1.53	1.73	1.4	2.14	1.18	0.97	1.42
Decile 3	1.85	1.52	2.26	2.25	1.84	2.77	1.5	1.26	1.8
Decile 4	2.12	1.75	2.57	2.52	2.05	3.08	1.87	1.57	2.23
Decile 5	2.17	1.79	2.63	2.76	2.26	3.37	1.77	1.48	2.11
Decile 6	2.51	2.08	3.03	2.53	2.07	3.1	2.02	1.7	2.4
Decile 7	2.79	2.31	3.36	3.11	2.56	3.79	2.27	1.92	2.69
Decile 8	3.43	2.86	4.11	3.77	3.11	4.58	2.29	1.93	2.71
Decile 9	3.86	3.22	4.61	4.81	3.98	5.8	2.52	2.14	2.98
Decile 10	6.2	5.22	7.36	6.71	5.58	8.06	3.71	3.16	4.35
Age	1.07	1.06	1.07	1.07	1.06	1.07	1.07	1.06	1.07

Gender male	2.16	2.01	2.31	2.14	2	2.29	2.11	1.97	2.26

Supplementary Table 4. Information Value (IV) Results

**Variable**	**IV**	**Interpretation**	**Comments**
Binned age × bmi	1.059556	Very Strong	Best predictor
Binned bmi	0.847075	Very Strong	Considered in model development
Binned age	0.704850	Very Strong	Considered in model development
Binned PRS SBP	0.215210	Medium	Considered in model development
Binned PRS DBP × PRS SBP	0.212097	Medium	Considered in model development
Binned PRS DBP	0.204780	Medium	Considered in model development
Binned PRS DBP × PRS PP	0.183856	Medium	Considered in model development
Binned PRS SBP × PRS PP	0.136822	Medium	Considered in model development
Gender	0.108220	Medium	Considered in model development
Binned PRS PP	0.107647	Medium	Considered in model development
Education	0.082677	Weak	Considered in model development

Training	0.069669	Weak	Considered in model development
Work	0.057945	Weak	Considered in model development
Alcohol Consumption	0.026486	Weak	Considered in model development
Steps	0.010237	Very Weak	Excluded from further analysis
Smoking Status	0.005127	Very Weak	Excluded from further analysis
Bad habits	0.000428	Very Weak	Excluded from further analysis

Supplementary Table 5. Model Performance and Hyperparameters

model	hyperparameters
**Dense NN**	{'n_layers': 2,<br>'dropout_rate': 0.18244376967320217,<br>'lr': 0.04737413260891494,<br>'optimizer': 'sgd',<br>'use_batchnorm': False,<br>'n_units_l0': 128,<br>'n_units_l1': 48}
**ResNet**	{'n_blocks': 3,<br>'dropout_rate': 0.3254987578191257,<br>'lr': 0.0002593698579846514,<br>'optimizer': 'adam',<br>'use_batchnorm': True,

	'n_units_block0': 192,<br>'n_units_block1': 160,<br>'n_units_block2': 64}
**LogisticRegression**	{'C': 0.9138588760239689,<br>'solver': 'saga',<br>'class_weight': {0: 1, 1: 24},<br>'l1_ratio': 0.20813503970565816}
**CatBoost**	{'n_estimators': 439, 'max_depth': 4, 'learning_rate': 0.024888393800043165}
**XGBoost**	{'n_estimators': 424,<br>'max_depth': 2,<br>'learning_rate': 0.020190210814371116,<br>'subsample': 0.614662967255552}
**LGBM**	{'n_estimators': 226,<br>'max_depth': 2,<br>'learning_rate': 0.02974302911032647,<br>'subsample': 0.7578910845312908}
**Dense NN with features from the questionnaire**	{'n_layers': 2,<br>'dropout_rate': 0.17880594781481066,<br>'lr': 0.010108223829122628,<br>'optimizer': 'sgd',<br>'use_batchnorm': True,<br>'n_units_l0': 128,<br>'n_units_l1': 128}

**RandomForest**	{'n_estimators': 912, 'max_depth': 4}
**Dense NN with only PRS**	{'n_layers': 3,<br>'dropout_rate': 0.12827344949067526,<br>'lr': 0.006093695158516269,<br>'optimizer': 'sgd',<br>'use_batchnorm': True,<br>'n_units_l0': 96,<br>'n_units_l1': 112,<br>'n_units_l2': 16}

Supplementary Table 6. Model SHAP Values for Feature Importance

Rank	Feature	Mean |SHAP|	Share, %	Mean SHAP	Std |SHAP|
1	**BMI**	0.009185	16.72	0.000981	0.007121
2	**Age * BMI**	0.008979	16.35	0.001003	0.011639
3	**Gender**	0.007716	14.05	0.000141	0.003618
4	**Age**	0.006955	12.66	0	0.008631
5	**PRS DBP**	0.005911	10.76	-0.000173	0.005961
6	**PRS SBP × PRS PP**	0.00478	8.7	-0.000844	0.005193
7	**Training**	0.002477	4.51	-0.000231	0.002498
8	**Education**	0.001854	3.37	3.40E-05	0.002798

9	**PRS DBP × PRS SBP**	0.001472	2.68	-1.00E-06	0.001978
10	**PRS SBP**	0.001244	2.27	2.10E-05	0.001601
11	**Alcohol Consumption**	0.001211	2.21	-0.000407	0.00126
12	**PRS PP**	0.001137	2.07	-4.00E-05	0.001562
13	**PRS DBP × PRS PP**	0.001033	1.88	0.00052	0.001189
14	**Work**	0.000975	1.77	9.70E-05	0.001245

Supplementary Table 7. Association of Hypertension Risk Scores with Clinical Outcomes Across Ancestry Groups

Group	N (cases/controls)	N (cases/controls), Female	DBP_beta+CI	DBP_p_raw	DBP_p_adj	SBP_beta+CI	SBP_p_raw	SBP_p_adj	PP_beta+CI	PP_p_raw	PP_p_adj
**Ashkenazi Jews**	746 (45/701)	301 (16/285)	0.3223 [0.03831; 0.6063]	0.0261	0.0356	0.5395 [0.1779; 0.9011]	0.0035	0.0047	0.2404 [-0.08127; 0.5622]	0.143	0.1787
**East Slavs and Mordvins**	101,317 (2,976/98,341)	56,373 (1,169/55,204)	0.4494 [0.4143; 0.4845]	0	0	0.5993 [0.5571; 0.6415]	0	0	0.3902 [0.3503; 0.4301]	0	0
Belarusians	5,464 (158/5,306)	3,119 (61/3,058)	0.4145 [0.2727; 0.5563]	0	0	0.6417 [0.4622; 0.8212]	0	0	0.4609 [0.2864; 0.6353]	0	0
Mordvins	659 (25/634)	382 (9/373)	0.5856 [0.2231; 0.948]	0.0015	0.0026	0.8535 [0.3502; 1.357]	0.0009	0.0015	0.2787 [-0.1216; 0.679]	0.1723	0.1989
Russians	79,142 (2,361/76,781)	43,957 (935/43,022)	0.4503 [0.411; 0.4896]	0	0	0.6045 [0.5571; 0.6519]	0	0	0.3981 [0.3532; 0.443]	0	0

Ukrainians	16,052 (432/15,620)	8,915 (164/8,751)	0.4431 [0.3441; 0.5421]	0	0	0.5345 [0.4229; 0.6461]	0	0	0.3242 [0.2197; 0.4288]	0	0
**North-Caucasus**	1,451 (13/1,438)	662 (4/658)	0.6256 [0.1758; 1.075]	0.0064	0.0096	0.7025 [0.06263; 1.342]	0.0314	0.0362	0.2398 [-0.3409; 0.8205]	0.4182	0.4481
**Siberia**	1,008 (37/971)	597 (20/577)	0.3851 [-0.02361; 0.7938]	0.0648	0.0747	0.6406 [0.2133; 1.068]	0.0033	0.0047	0.432 [0.0473; 0.8167]	0.0277	0.0462
Buryats,Hamnigan,Mongols	338 (14/324)	203 (9/194)	0.5836 [-0.02961; 1.197]	0.0621	0.0747	0.9563 [0.1966; 1.716]	0.0136	0.017	0.5111 [-0.1504; 1.173]	0.1299	0.1772
Dolgans,Yakuts	642 (22/620)	376 (10/366)	0.1354 [-0.4232; 0.6939]	0.6348	0.6348	0.4766 [-0.07685; 1.03]	0.0915	0.098	0.4262 [-0.06977; 0.9221]	0.0921	0.1382
**Volga-Ural region**	5,381 (124/5,257)	2881 (50/2,831)	0.4883 [0.3195; 0.6571]	0	0	0.5961 [0.3835; 0.8087]	0	0	0.2896 [0.0879; 0.4913]	0.0049	0.0105
Chuvash	1,122 (27/1,095)	629 (9/620)	0.2384 [-0.1384; 0.6153]	0.2149	0.2303	0.2999 [-0.1605; 0.7602]	0.2017	0.2017	0.0616 [-0.3832; 0.5064]	0.786	0.786
Tatars,Volga-Tatars,Mishar-Tatars,Kryashens	3,850 (85/3,765)	2006 (34/1,972)	0.5171 [0.3089; 0.7254]	0	0	0.679 [0.4183; 0.9398]	0	0	0.3855 [0.1403; 0.6308]	0.0021	0.0052
**Western-Asia**	2,178 (34/2,144)	924 (10/914)	0.7441 [0.3593; 1.129]	0.0002	0.0003	0.7973 [0.3917; 1.203]	0.0001	0.0002	0.4652 [0.07304; 0.8574]	0.0201	0.0376
Armenians,Hemshins	1,900 (28/1,872)	835 (10/825)	0.82 [0.4051; 1.235]	0.0001	0.0002	1.009 [0.5596; 1.459]	0	0	0.7012 [0.2667; 1.136]	0.0016	0.0047
